# Urinary phytoestrogen excretion and prostate cancer risk: a nested case–control study in the Multiethnic Cohort

**DOI:** 10.1038/sj.bjc.6605137

**Published:** 2009-06-16

**Authors:** S-Y Park, L R Wilkens, A A Franke, L Le Marchand, K K Kakazu, M T Goodman, S P Murphy, B E Henderson, L N Kolonel

**Affiliations:** 1 Epidemiology Program, Cancer Research Center of Hawaii, University of Hawaii, Honolulu, HI 96813, USA; 2 Natural Products and Cancer Biology Program, Cancer Research Center of Hawaii, University of Hawaii, Honolulu, HI 96813, USA; 3 Department of Preventive Medicine, Keck School of Medicine, University of Southern California, Los Angeles, CA 90089, USA

**Keywords:** phytoestrogens, urinary excretion, prostate neoplasms, case–control study, multiethnic population

## Abstract

**Background:**

Phytoestrogens are of special interest in prostate cancer research because populations in Asia with a high consumption of phytoestrogens have a lower incidence of the disease than comparable populations in Western countries.

**Methods:**

This case–control study is nested within a large multiethnic cohort in Hawaii and California. Urine samples were analysed for daidzein, genistein, equol, and enterolactone among 249 incident prostate cancer cases and 404 controls matched on age, race/ethnicity, date/time of specimen collection, and fasting status.

**Results:**

The median excretion of daidzein was 0.173 nmol mg^−1^ creatinine in cases and 0.291 in controls (*P*=0.01), and the median excretion of genistein was 0.048 in cases and 0.078 in controls (*P*=0.05). An inverse association was seen for daidzein overall (odds ratio for the highest *vs* lowest quintile=0.55, 95% confidence interval=0.31–0.98, *P*_trend_=0.03) and seemed to apply to localized (*P*_trend_=0.08) as well as advanced or high-grade cancer (*P*_trend_=0.09). This association was consistent across the four ethnic groups examined. Although the relationship was weaker for genistein, the odds ratios and trends were similarly inverse. Urinary excretion of equol and enterolactone was not significantly related to prostate cancer risk.

**Conclusion:**

Our findings suggest that high intake of isoflavones, as reflected by urinary excretion of daidzein and genistein, may be protective against prostate cancer.

The incidence of prostate cancer is low in Asia, particularly China and Japan, compared with Western countries including the United States, Canada, and parts of Europe (Parkin *et al*, 2002). Diet has been suggested as the cause of this international difference, and phytoestrogens are of special interest, as their consumption as in foods such as soy is much higher in Asia than in Western countries (Adlercreutz, 2002; Messina, 2003). Phytoestrogens can be divided into two main classes: isoflavones primarily found in soy and lignans found in a wide range of plants (Magee and Rowland, 2004). Daidzein and genistein are major soy isoflavones and equol is produced from daidzein by intestinal bacteria. The mammalian lignans, enterodiol and enterolactone, are converted from plant precursors by the gut bacteria in the colon (Kurzer and Xu, 1997; Adlercreutz, 2002). Phytoestrogens may play a role in prostate cancer through a variety of mechanisms, not all related to estrogenic or antiestrogenic effects. Phytoestrogens act as free radical scavengers and have effects on prostate-specific antigen (PSA) production, androgen activity, apoptosis, angiogenesis, sex hormone binding globulin, and gene expression (Magee and Rowland, 2004). Although experimental studies have supported such mechanisms and some epidemiologic studies found a protective effect of dietary phytoestrogens against prostate cancer, including a recent meta-analysis (Yan and Spitznagel, 2005), the evidence from observational studies is not consistent (Ganry, 2005).

Urinary excretion of phytoestrogens has been shown to be a valid biomarker of phytoestrogen intake (Maskarinec *et al*, 1998; Seow *et al*, 1998; Chen *et al*, 1999; Lampe, 2003). Few studies, however, have examined urinary phytoestrogens in relation to prostate cancer risk (Low *et al*, 2006; Ward *et al*, 2008). In this case–control study nested within a multiethnic cohort, we assessed the effects of urinary excretion of daidzein, genistein, equol, and enterolactone on prostate cancer risk.

## Materials and methods

### Study population and data collection

We carried out a nested case–control study within the Multiethnic Cohort Study, established in Hawaii and Los Angeles, California, in 1993–1996 (Kolonel *et al*, 2000b). The study was approved by the review boards of the University of Hawaii and the University of Southern California. More than 215 000 adults aged 45–75 years entered the cohort by completing a detailed questionnaire on diet and lifestyle factors, which targeted five ethnic/racial groups: African Americans, Native Hawaiians, Japanese Americans, Latinos, and Whites. A prospective biospecimen subcohort was developed, largely between 2001 and 2006, when cohort members who gave informed consent to participate provided a blood and/or urine sample. An overnight (Hawaii) or a first morning (Los Angeles) urine sample was preferentially collected; a spot urine was requested otherwise.

### Case ascertainment and control selection

Identification of incident, invasive prostate cancer cases was accomplished through linkage to the population-based cancer registries covering Hawaii and California, which are members of the Surveillance, Epidemiology and End Results Program of the National Cancer Institute. Deaths were identified by linkage to death-certificate files in Hawaii and California, as well as to the National Death Index. Advanced prostate cancers were defined as invasive cancers that were regional or metastatic (not localized), whereas high-grade prostate cancers were based on Gleason score (⩾7 categorized as poorly differentiated). During the follow-up period (mean follow-up, 1.9 years), 467 eligible prostate cancer cases were identified. Two controls for each cases were randomly selected from the men who were alive and free of prostate cancer at the age of the case's diagnosis, by matching for geographic location (Hawaii or California), race/ethnicity, birth year (±1 year), date (±6 months) and time (±2 h) of specimen collection, and fasting hours (0–<6, 6–<8, 8–<10, and 10+ h). Out of these triplets, 288 cases and 540 controls had an overnight or first morning urine sample as required for phytoestrogen assessment. This included 249 matched sets, 94 with one control and 155 with two controls (249 cases and 404 controls). The matching on area ensured that cases and controls with a matched set used the same urine collection regime (i.e., overnight in Hawaii or first morning in California).

### Laboratory assays

Daidzein, genistein, equol, and enterolactone were analysed by HPLC with electrospray ionization tandem mass spectrometry using isotopically labelled internal standards of all four analytes (Universities of St. Andrews, UK, and Helsinki, Finland), similar to our earlier reports (Franke *et al*, 2002; Blair *et al*, 2003; Maskarinec *et al*, 2008). Limits of quantitation (LOQ) were 1 nM for daidzein (all subjects>LOQ) and genistein (31<LOQ), 2 nM for equol (238<LOQ), and 5 nM for enterolactone (4<LOQ). Between-day coefficients of variation were 4–10% for daidzein, 5–12% for genistein, 5–18% for equol, and 5–15% for enterolactone, whereas intra-day variation was less than or equal to half of these values.

### Statistical analyses

The differences in means and proportions between cases and controls for several characteristics were tested, respectively, by the *t*-test and χ^2^ test. As the distributions of urinary phytoestrogens were skewed to the right, medians were tested by the Wilcoxon test for differences between cases and controls. The sum of daidzein and genistein was calculated as an estimate of main nonmetabolized isoflavones and the sum of daidzein, genistein, and equol as an estimate of major total isoflavonoids. Subjects were divided into quantiles based on the overall distribution of urinary phytoestrogens in both cases and controls. For equol, the subjects with values below the LOQ (*n*=238, 36%) were assigned to the lowest quantile, and the others were evenly divided into two groups. Conditional logistic regression models were used to estimate odds ratios (ORs) and 95% confidence intervals (CIs) of prostate cancer for the upper quantile groups compared with the lowest quantile as a reference group. To test for linear trend, a variable assigned the median of the appropriate quantile was entered into the model as a continuous variable. The significance of the trend was based on the Wald statistic for the trend variable. Matched sets were used as strata in the conditional logistic models, which accounted for the matching criteria. The ORs were further adjusted for age at specimen collection and fasting hours before specimen collection as continuous variables to account for any possible systematic differences within matched sets, as well as family history of prostate cancer (yes *vs* no), body mass index (BMI, weight/(height)^2^; overweight and obese *vs* normal), and education (continuous).

The models were run separately for localized but not high-grade cases and their matched controls, and for advanced and/or high-grade cases and their matched controls. The test of heterogeneity across cancer stage/grade was performed by fitting the simultaneous conditional logistic regressions for localized and advanced cancers and testing an interaction between event type and exposure variable by a likelihood ratio test. In an ethnic-specific analysis, Native Hawaiians were not included because the number of cases was too small (*n*=6). Tests for interaction between urinary phytoestrogen excretion and ethnicity in relation to prostate cancer risk were based on likelihood ratios between models with and without interaction terms. Separate analyses were performed excluding controls with high PSA values (>4 ng ml^−1^), stratifying by BMI category and smoking status, and restricting to equol producers, defined by an equol to daidzein ratio of greater than 0.018 (Setchell and Cole, 2006). These analyses used unconditional logistic regression to maximize the number of subjects available and adjusted for the matching criteria variables. Statistical significance was considered at *P*<0.05 and all tests were two sided.

## Results

There was no substantial difference in education, BMI, physical activity, and family history of prostate cancer between cases and controls (Table 1). The average time from specimen collection to the case's diagnosis date was 1.6 years in our analysis set and was not different between cases and controls; this is less than the overall 1.9 years given earlier, as spot urines were collected from the earliest participants in the repository, whereas the first morning/overnight collection regimes were established later. The median excretion of daidzein and genistein was lower in cases than in controls (*P*=0.01 and 0.05, respectively), whereas the median excretion of equol and enterolactone did not differ between the two groups.

The associations between urinary excretion of phytoestrogens and prostate cancer risk are presented in Table 2. Urinary excretion of daidzein was inversely associated with prostate cancer risk: OR for the highest *vs* lowest quintile=0.55, 95% CI=0.31–0.98, and *P*_trend_=0.03. There was a nonsignificant inverse trend for genistein (*P*_trend_=0.09), for daidzein+genistein combined (*P*_trend_=0.07), and for equol (*P*_trend_=0.08). When we limited the analysis to equol producers (51%, 144 cases and 189 controls), no significant association was found (*P*_trend_=0.64). The results remained similar when excluding controls with a high PSA value (>4 ng ml^−1^, *n*=62, data not shown).

Table 3 shows the relationship between phytoestrogens and prostate cancer by stage/grade. For daidzein, an inverse linear trend is suggested for both localized and advanced or high-grade tumours, although the *P* values for trend (0.08 and 0.09, respectively) are not quite statistically significant. For genistein, the ORs for the highest tertiles were also below 1.00 for both localized and advanced or high-grade disease, though the trends were not statistically significant. Similar patterns were seen for the combinations of daidzein, genistein, and equol. There was no statistically significant heterogeneity in ORs across stage/grade, except for enterolactone (*P*_interaction_=0.02), which showed a nonsignificant increased risk in the third tertile for advanced or high-grade tumours.

The number of cases for racial/ethnic-specific analyses was limited, so we could only dichotomize the urinary excretion at the median. Table 4 shows the results for four ethnic groups (the number of cases in Native Hawaiians was too few for a separate analysis). None of the interactions were significant. The association of daidzein with prostate cancer risk was inverse for all four ethnic groups, although only Latinos showed a statistically significant reduction. For genistein, an inverse association was seen for two of the four groups.

In analyses stratified by BMI category or smoking status, there was no statistically significant association or interaction (data not shown). However, the number of cases in each cell, particularly for the obese group and the current smokers, were too small for stable estimates.

## Discussion

In this case–control study nested within a multiethnic cohort, we found an inverse association between urinary daidzein excretion and prostate cancer risk. A suggestive inverse trend for daidzein was seen for localized, as well as advanced or high-grade cancer, and across the four ethnic groups examined. Urinary excretion of genistein, equol, and enterolactone was not significantly related to prostate cancer risk.

In earlier observational studies, dietary intake of isoflavones or soy product consumption was inversely associated with prostate cancer risk in some (Severson *et al*, 1989; Jacobsen *et al*, 1998; Strom *et al*, 1999; Kolonel *et al*, 2000a; Lee *et al*, 2003; Sonoda *et al*, 2004; Hedelin *et al*, 2006a; Heald *et al*, 2007; Kurahashi *et al*, 2007; Nagata *et al*, 2007), but not all reports (Sung *et al*, 1999; Villeneuve *et al*, 1999; Allen *et al*, 2004; Nomura *et al*, 2004; Low *et al*, 2006; Hedelin *et al*, 2006b). A meta-analysis of soy food consumption and prostate cancer, including two cohort and six case–control studies, reported a 30% overall risk reduction (Yan and Spitznagel, 2005). There are only a few studies that have examined biomarkers of isoflavone intake in relation to prostate cancer risk. In a nested case–control study of Japanese men, Ozasa *et al* (2004) found that high serum levels of genistein, daidzein, and equol were associated with a decreased risk of prostate cancer. Two reports from the European Prospective Investigation of Cancer-Norfolk Study (Low *et al*, 2006; Ward *et al*, 2008) did not find any association of either serum or urinary isoflavones with prostate cancer risk. Also, a case–control study in Scottish men reported no association between serum isoflavones and prostate cancer risk (Heald *et al*, 2007).

The extent of urinary isoflavone excretion would be expected to be higher and the ranges wider in our study population than in the populations of most Western countries, because 29% of the subjects in our study were Japanese Americans who consume relatively high amounts of soy products. In our controls, the median values (0.29 nmol mg^−1^ creatinine for daidzein and 0.08 for genistein) were similar to those reported for the US adult (⩾20 years) population (0.23 for daidzein and 0.08 for genistein) (Valentin-Blasini *et al*, 2005), whereas they were much less than in the Singapore population where the medians were 1.37 for daidzein and 0.73 for genistein (Seow *et al*, 1998). However, there were substantial differences across the racial/ethnic groups, ranging from 0.13 nmol mg^−1^ creatinine in African Americans to 1.12 in Japanese Americans for daidzein, and from 0.03 in African Americans to 0.74 in Japanese Americans for genistein confirming our earlier findings in women (Maskarinec *et al*, 1998).

In this study, the reason that the effect of genistein on prostate cancer risk was not as strong as that of daidzein is not clear, although the correlation between these two isoflavones in urine was high (*r*=0.90, *P*<0.001). In serum or plasma, genistein concentrations are generally higher than daidzein, whereas the opposite occurs in urine (Lampe, 2003). This suggests that the metabolism and excretion of these isoflavones are not identical and explains the higher bioavailability of genistein compared with daidzein (Franke *et al*, 2009). As urinary excretion of daidzein and genistein reflect the corresponding circulating levels equally well, that is, plasma/urine ratios of both daidzein and genistein are constant within and between individuals (Franke *et al*, 2004), daidzein may have a greater potential to prevent prostate cancer than does genistein. The enhanced rate of glucuronidation of estrogen by daidzein and its metabolites may explain the protective effect of soy isoflavones against hormonal cancers (Pfeiffer *et al*, 2005). Daidzein may also be a surrogate for the effects of its metabolite equol that was shown to specifically bind 5alpha-dihydrotestosterone (DHT) and thereby inhibit DHT's stimulation of prostate cell growth (Lund *et al*, 2004), although we did not observe any association between equol and prostate cancer risk in this study.

Previously, in a prospective analysis of the entire multiethnic cohort (*n*=82 483 including 4404 cases), we found a modest risk reduction for total prostate cancer in men with highest soy intake (relative risk (RR)=0.90, 95% CI=0.80–1.01) and a stronger effect for advanced or high-grade prostate cancer (RR=0.78, 95% CI=0.62–0.98) (Park *et al*, 2008). However, we found no significant association with estimated intakes of genistein or daidzein. Although we used our valid quantitative food frequency questionnaire and comprehensive food composition table, there might be more measurement error in these estimates than in the biomarker assays. Furthermore, the food composition table might not capture all sources of phytoestrogens in the diet. However, urinary excretion of isoflavones has been shown to have a linear dose response with dietary intake of isoflavones in feeding studies and observational studies (Lampe, 2003). In earlier studies, urinary isoflavone excretion was significantly correlated with usual isoflavone intake in women from a multiethnic population in Hawaii (*r*=0.31, *P*<0.01) (Maskarinec *et al*, 1998), and with usual soy food consumption among women in Shanghai (*r*=0.50, *P*<0.001) (Chen *et al*, 1999).

Equol is a product of intestinal bacterial metabolism of daidzein, and it has estrogenic activity (Setchell *et al*, 2002). In the studies in European countries, urinary and/or serum equol were not related to prostate cancer (Low *et al*, 2006; Heald *et al*, 2007; Ward *et al*, 2008), whereas the study among Japanese men where soy intake was much higher found an inverse association between serum equol and prostate cancer risk (Ozasa *et al*, 2004). Whether we restricted the analyses to equol producers or not, we observed no relation of urinary equol excretion with prostate cancer risk.

One case–control study found a 60% reduction in prostate cancer risk associated with a high concentration of serum enterolactone (Heald *et al*, 2007), but no relationship was seen in other studies that measured enterolactone in serum, plasma, or urine (Stattin *et al*, 2002, 2004; Kilkkinen *et al*, 2003; Low *et al*, 2006; Hedelin *et al*, 2006b). One possible reason for the lack of associations in earlier studies is that enterolactone levels were too low for a protective effect to be observed (Heald *et al*, 2007). In our study, urinary enterolactone excretion (median=1.09 nmol mg^−1^ creatinine in controls) was lower than in the study of Low *et al* in the UK (mean=2.06 nmol mg^−1^ creatinine in controls) (Low *et al*, 2006) and similar to that in the US adult population (median=0.96 nmol mg^−1^ creatinine) (Valentin-Blasini *et al*, 2005). Thus, our failure to observe an association with prostate cancer risk was possibly due to the low excretion of enterolactone in our population.

Our prospective design reduced the possibility that the disease influenced either dietary intake or urinary excretion of phytoestrogens. Furthermore, when we excluded controls with elevated PSA levels (>4 ng ml^−1^) (some of whom may have had undiagnosed prostate tumours), the results did not change. The different ethnic groups led to a wide range in concentration of measured analytes thereby minimizing the risk of not being able to observe effects within or between the low and high phytoestrogen exposed groups. Although this is a relatively small study, we were able to detect as significant a linear trend in ORs from 1 to 0.55 across daidzein quintiles (*P*=0.03). The minimum detectable OR in quintile 5, assuming a linear trend in ORs, a power of 80% and a critical value of 0.05, was 0.56. Therefore, our power to detect a more modest OR of 0.72, as found for genistein, was suboptimal.

However, certain limitations must be considered in this study. Urinary isoflavones reflect short term (24–48 h) rather than long-term dietary intake (Franke *et al*, 2004). Furthermore, only one overnight or first morning urine sample was collected and, thus, might not represent usual intake, although three measurements of urinary isoflavone excretion over a 3-week period were in acceptable agreement in a pilot study that was conducted among 20 individuals (unpublished). As dietary intake was not assessed at the time of urine collection, we were not able to look at how well urinary isoflavone excretion correlated with usual intake of isoflavones among our subjects. However, a study using a one-time urine sample reported that urinary isoflavones represented usual intakes of dietary isoflavone as measured by 24 h dietary recall in US adults (Chun *et al*, 2009). Follow-up time (average 1.6 years) was relatively short, and thus further studies in this cohort will be of interest when more follow-up has accrued. Another limitation is reduced statistical power for the subgroup analyses due to the smaller number of cases.

In conclusion, high urinary excretion of the soy isoflavones, especially daidzein, seemed to be protective against prostate cancer in a multiethnic population in which the variation of isoflavones in urine was wide.

## Figures and Tables

**Table 1:** Characteristics of cases and controls^a^

	**Cases (*n*=249)**	**Controls (*n*=404)**	***P*-value^b^**
Age at specimen collection (years)	69.2±6.7^c^	69.2±6.9	0.96
Time between specimen collection and case diagnosis (years)	1.6±1.5	1.5±1.4	0.49
Fasting hours before specimen collection	12.7±3.3	13.0±3.2	0.32
Education (years)	14.0±2.8	14.0±2.9	0.71
Body mass index (kg m^−2^)	25.8±3.9	26.0±3.7	0.45
METS of activity per day	1.64±0.28	1.64±0.30	0.93
Family history of prostate cancer (%)	10.0	7.7	0.29
			
*Ethnicity* (%)			
African Americans	37.0^d^	32.9	0.80
Native Hawaiians	2.4	2.7	
Japanese Americans	28.1	30.2	
Latinos	17.7	16.8	
Whites	14.9	17.3	
			
*Urinary phytoestrogens* e			
Daidzein	0.173 (0.057, 0.652)^f^	0.291 (0.084, 1.307)	0.01
Genistein	0.048 (0.013, 0.303)	0.078 (0.016, 0.495)	0.05
Equol	0.005 (<LOQ^g^, 0.017)	0.004 (<LOQ, 0.013)	0.42
Daidzein+Genistein	0.235 (0.081, 1.015)	0.428 (0.112, 1.996)	0.01
Daidzein+Genistein+Equol	0.257 (0.094, 1.194)	0.438 (0.118, 2.112)	0.03
Enterolactone	1.042 (0.273, 2.327)	1.094 (0.401, 2.253)	0.97

a Matching for geographic location (Hawaii or California), race/ethnicity, birth year (±1 year), date (±6 months) and time (±2 h) of specimen collection, and fasting hours (0–<6, 6–<8, 8–<10, and 10+ h).

b Tested by *t*-test (means), χ^2^ test (percentages), and Wilcoxon rank-sum test (medians).

c Mean±s.d.

d Column percentage.

e Expressed as nmol mg^−1^ creatinine.

f Median (25th, 75th percentile).

g <LOQ, less than limit of quantitation.

**Table 2:** Associations of urinary phytoestrogen excretion with prostate cancer risk^a^

	**Q1**	**Q2**	**Q3**	**Q4**	**Q5**	***P*-value for linear trend**
Daidzein	⩽0.053^b^	0.053–⩽0.151	0.151–⩽0.386	0.386–⩽1.630	>1.630	
Cases/controls	59/71	57/75	48/81	45/86	40/91	
OR (95% CI)	1.00	1.03 (0.61–1.74)	0.83 (0.50–1.38)	0.74 (0.44–1.25)	0.55 (0.31–0.98)	0.03
						
Genistein	⩽0.009	0.009–⩽0.038	0.038–⩽0.126	0.126–⩽0.729	>0.729	
Cases/controls	54/76	62/70	46/87	45/85	42/89	
OR (95% CI)	1.00	1.48 (0.88–2.49)	0.91 (0.53–1.55)	0.99 (0.59–1.69)	0.72 (0.40–1.31)	0.09
						
Equol	⩽0.001	0.001–⩽0.010	>0.010			
Cases/controls	90/148^c^	70/137	89/119			
OR (95% CI)	1.00	0.89 (0.58–1.37)	1.32 (0.84–2.08)			0.08
						
Equol (producers only)^d^	⩽0.005	0.005–⩽0.023	>0.023			
Cases/controls	47/64	47/64	50/61			
OR (95% CI)	1.00	1.05 (0.59–1.85)	1.32 (0.72–2.42)			0.64
						
Daidzein+Genistein	⩽0.069	0.069–⩽0.202	0.202–⩽0.522	0.522–⩽2.565	>2.565	
Cases/controls	56/74	62/69	44/86	45/87	42/88	
OR (95% CI)	1.00	1.38 (0.83–2.30)	0.74 (0.44–1.24)	0.83 (0.49–1.39)	0.63 (0.35–1.14)	0.07
						
Daidzein+Genistein+Equol	⩽0.077	0.077–⩽0.213	0.213–⩽0.549	0.549–⩽2.773	>2.773	
Cases/controls	56/74	58/73	47/84	44/86	44/87	
OR (95% CI)	1.00	1.27 (0.76–2.21)	0.84 (0.51–1.40)	0.86 (0.51–1.44)	0.74 (0.42–1.30)	0.19
						
Enterolactone	⩽0.227	0.227–⩽0.798	0.798–⩽1.493	1.493–⩽2.667	>2.667	
Cases/controls	57/74	47/83	38/92	55/77	52/78	
OR (95% CI)	1.00	0.71 (0.42–1.21)	0.58 (0.34–0.98)	0.97 (0.57–1.66)	0.98 (0.58–1.66)	0.44

a Matching for geographic location (Hawaii or California), race/ethnicity, birth year (±1 year), date (±6 months) and time (±2 h) of specimen collection, and fasting hours (0–<6, 6–<8, 8–<10, and 10+ h). The models were adjusted for age at specimen collection and fasting hours as continuous variables, as well as family history of prostate cancer, BMI, and education.

b Expressed as nmol mg^−1^ creatinine.

c Subjects with values below the limit of quantitation.

d Unconditional logistic regression was used with adjustment for the matching criteria and the same covariates as in conditional logistic models.

**Table 3:** Associations of urinary phytoestrogen excretion with prostate cancer risk by tumour characteristics^a^

	**T1**	**T2**	**T3**	***P*-value for linear trend**	***P*-value for heterogeneity^b^**
Daidzein	⩽0.115^c^	0.115–⩽0.569	>0.569		
					
*Localized* d					0.83
Cases/controls	53/70	46/66	36/84		
OR (95% CI)	1.00	0.93 (0.55–1.59)	0.59 (0.31–1.11)	0.08	
					
*Advanced or high grade*					
Cases/controls	26/33	24/37	18/33		
OR (95% CI)	1.00	0.98 (0.41–2.36)	0.46 (0.17–1.27)	0.09	
					
Genistein	⩽0.024	0.024–⩽0.200	>0.200		
*Localized*					1.00
Cases/controls	52/68	44/69	39/83		
OR (95% CI)	1.00	1.01 (0.59–1.73)	0.74 (0.38–1.42)	0.32	
					
*Advanced or high grade*					
Cases/controls	31/34	18/40	47/28		
OR (95% CI)	1.00	0.53 (0.23–1.20)	0.53 (0.21–1.37)	0.37	
					
Equol	⩽0.001	0.001–⩽0.010	>0.010		
*Localized*					0.66
Cases/controls	50/85^e^	34/63	51/72		
OR (95% CI)	1.00	1.10 (0.60–2.03)	1.38 (0.75–2.54)	0.29	
*Advanced or high grade*					
Cases/controls	22/27^e^	21/48	25/27		
OR (95% CI)	1.00	0.54 (0.22–1.31)	1.17 (0.42–3.23)	0.19	
					
Daidzein+Genistein	⩽0.150	0.150–⩽0.799	>0.799		
*Localized*					0.88
Cases/controls	54/69	44/66	37/85		
OR (95% CI)	1.00	0.85 (0.49–1.48)	0.57 (0.30–1.09)	0.10	
					
*Advanced or high grade*					
Cases/controls	28/34	23/37	17/31		
OR (95% CI)	1.00	1.04 (0.45–2.38)	0.44 (0.16–1.18)	0.07	
					
Daidzein+Genistein+Equol	⩽0.159	0.159–⩽0.847	>0.847		
*Localized*					0.96
Cases/controls	54/67	43/69	38/84		
OR (95% CI)	1.00	0.84 (0.49–1.45)	0.61 (0.32–1.16)	0.16	
					
*Advanced or high grade*					
Cases/controls	28/37	22/35	18/30		
OR (95% CI)	1.00	0.99 (0.44–2.23)	0.51 (0.19–1.34)	0.14	
					
Enterolactone	⩽0.616	0.616–⩽1.771	>1.771		
*Localized*					0.02
Cases/controls	51/71	43/85	41/64		
OR (95% CI)	1.00	0.79 (0.46–1.36)	0.87 (0.47–1.60)	0.74	
*Advanced or high grade*					
Cases/controls	21/31	16/36	31/35		
OR (95% CI)	1.00	0.76 (0.31–1.87)	1.60 (0.74–3.50)	0.12	

a Matching for geographic location (Hawaii or California), race/ethnicity, birth year (±1 year), date (±6 months) and time (±2 h) of specimen collection, and fasting hours (0–<6, 6–<8, 8–<10, and 10+ h). The models were adjusted for age at specimen collection and fasting hours as continuous variables, as well as family history of prostate cancer, BMI, and education. Forty-six cases were missing stage/grade.

b The *P* for heterogeneity between localized and advanced or high grade cancers is based on the likelihood ratio test.

c Expressed as nmol mg^−1^ creatinine.

d Localized but not of high grade.

e Subjects with values below the limit of quantitation.

**Table 4:** Associations of urinary phytoestrogen excretion with prostate cancer risk by race/ethnicity^a^

	**African Americans**		**Japanese Americans**		**Latinos**		**Whites**		
	**Cases/controls**	**OR (95% CI)**	**Cases/controls**	**OR (95% CI)**	**Cases/controls**	**OR (95% CI)**	**Cases/controls**	**OR (95% CI)**	***P* for interaction^b^**
*Daidzein*									
⩽0.234^c^	68/87	1.00	19/27	1.00	31/33	1.00	22/30	1.00	
>0.234	24/46	0.75 (0.39–1.45)	51/95	0.71 (0.35–1.42)	13/35	0.38 (0.16–0.92)	15/40	0.47 (0.19–1.20)	0.65
									
Genistein									
⩽0.067	64/91	1.00	16/26	1.00	30/37	1.00	23/31	1.00	
>0.067	28/42	1.28 (0.69–2.38)	54/96	0.92 (0.44–1.95)	14/31	0.52 (0.23–1.21)	14/39	0.42 (0.17–1.06)	0.34
									
Equol									
⩽0.004	53/77	1.00	33/66	1.00	17/35	1.00	12/26	1.00	
>0.004	39/56	1.06 (0.53–2.11)	37/56	1.62 (0.80–3.28)	27/33	2.14 (0.76–6.03)	25/44	1.27 (0.48–3.37)	0.85
									
Daidzein+Genistein									
⩽0.326	68/90	1.00	18/24	1.00	31/32	1.00	23/31	1.00	
>0.326	24/43	0.84 (0.43–1.64)	52/98	0.63 (0.31–1.29)	13/36	0.37 (0.16–0.87)	14/39	0.41 (0.16–1.10)	0.54
									
Daidzein+Genistein									
+Equol									
⩽0.335	68/91	1.00	18/23	1.00	31/32	1.00	21/32	1.00	
>0.335	24/42	0.90 (0.46–1.76)	52/99	0.61 (0.30–1.26)	13/36	0.32 (0.13–0.80)	16/38	0.56 (0.21–1.46)	0.55
									
Enterolactone									
⩽1.091	44/63	1.00	47/72	1.00	19/35	1.00	12/23	1.00	
>1.091	48/70	1.02 (0.54–1.94)	23/50	0.67 (0.34–1.31)	25/33	1.76 (0.74–4.19)	25/47	1.17 (0.47–2.92)	0.33

a Matching for geographic location (Hawaii or California), race/ethnicity, birth year (±1 year), date (±6 months) and time (±2 h) of specimen collection, and fasting hours (0–<6, 6–<8, 8–<10, and 10+ h). The models were adjusted for age at specimen collection and fasting hours as continuous variables, as well as family history of prostate cancer, BMI, and education.

b The *P* for interaction between urinary phytoestrogen excretion and ethnicity is based on the likelihood ratio test.

c Expressed as nmol mg^−1^ creatinine.
